# Accurate calculation of the absolute free energy of binding for drug molecules†
†Electronic supplementary information (ESI) available. See DOI: 10.1039/c5sc02678d


**DOI:** 10.1039/c5sc02678d

**Published:** 2015-09-25

**Authors:** Matteo Aldeghi, Alexander Heifetz, Michael J. Bodkin, Stefan Knapp, Philip C. Biggin

**Affiliations:** a Structural Bioinformatics and Computational Biochemistry , Department of Biochemistry , University of Oxford , South Parks Road , Oxford , OX1 3QU , UK . Email: philip.biggin@bioch.ox.ac.uk ; Fax: +44 (0)1865 613238 ; Tel: +44 (0)1865 613305; b Evotec (U.K.) Ltd , 114 Innovation Drive, Milton Park , Abingdon , Oxfordshire OX14 4RZ , UK; c Structural Genomics Consortium , Nuffield Department of Clinical Medicine , University of Oxford , Old Road Campus Research Building, Roosevelt Drive , Oxford OX3 7DQ , UK; d Target Discovery Institute , Nuffield Department of Clinical Medicine , University of Oxford , Roosevelt Drive , Oxford OX3 7BN , UK

## Abstract

Accurate prediction of binding affinities has been a central goal of computational chemistry for decades, yet remains elusive. Despite good progress, the required accuracy for use in a drug-discovery context has not been consistently achieved for drug-like molecules. Here, we perform absolute free energy calculations based on a thermodynamic cycle for a set of diverse inhibitors binding to bromodomain-containing protein 4 (BRD4) and demonstrate that a mean absolute error of 0.6 kcal mol^–1^ can be achieved. We also show a similar level of accuracy (1.0 kcal mol^–1^) can be achieved in pseudo prospective approach. Bromodomains are epigenetic mark readers that recognize acetylation motifs and regulate gene transcription, and are currently being investigated as therapeutic targets for cancer and inflammation. The unprecedented accuracy offers the exciting prospect that the binding free energy of drug-like compounds can be predicted for pharmacologically relevant targets.

## Abstract 2

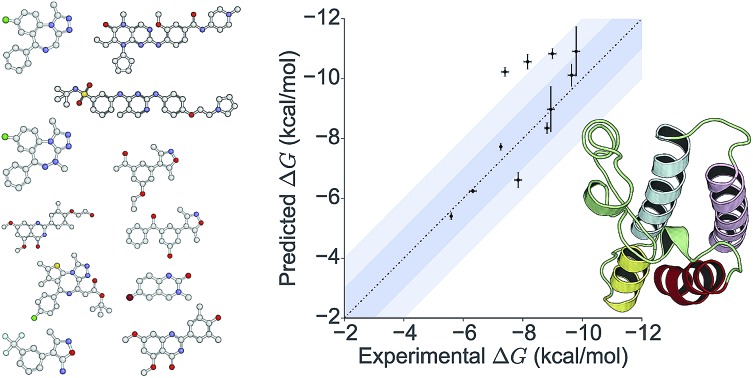
Free energy calculations based on molecular dynamics and thermodynamic cycles accurately reproduce experimental affinities of diverse bromodomain inhibitors.

## Introduction

One of the “holy grails” of computational drug design is the accurate prediction of the affinity of a drug for its target protein. Despite the development of pharmacologically active molecules being a multifactorial optimization problem, where other considerations too, such as bioavailability and toxicity, play an important role, high affinity of a compound for its intended biological target is a necessary requirement for achieving a potent, selective and ultimately efficacious drug. Unfortunately, even when structural information is available, solvent effects, conformational changes of the protein and/or the ligand and entropy–enthalpy compensation make the rationalization of the ligand–macromolecule association process a very complex task.^1^,^2^ However, thanks to important advances in theory and computing, particularly in the last decade, the prediction of binding affinities using physics-based computer simulations holds promise^3^,^4^ to achieve reliable binding energies estimates by naturally taking into account complicating effects due to the discrete nature of solvent and entropy changes upon binding.

Alchemical free energy calculations and steered methods based on all-atom molecular dynamics (MD) simulation in explicit solvent are the typical approaches that operate at the highest level of theoretical rigor and that are also accessible to current typical levels of computational power. Alchemical methods, often also referred to as free energy perturbation (FEP), are based on a non-physical thermodynamic cycle, where the binding free energy is computed as the sum of multiple steps during which the ligand is “inserted” or “removed” from different environments, such as a bound and unbound state.^5^ Steered or pulling method approaches follow instead a physical pathway, by applying a force that pulls the ligand away from the protein.^6^ This is typically achieved either with non-equilibrium simulations using the Jarzynski relationship,^7^–^9^ or by harmonically restraining the ligand at different distances from the binding pocket and then computing a potential of mean force.^5^,^10^,^11^ Alternative popular approaches include endpoint methods that involve implicit solvent post-processing of explicit-solvent simulations, such as molecular mechanics with Poisson–Boltzmann or generalized Born and surface area (MM/PBSA and MM/GBSA) methods.^12^–^15^ Another promising approach is metadynamics^16^ with a funnel-shaped restraining potential, where biasing energies are added in order to sample multiple binding events.^17^

Absolute binding free energies have been calculated with alchemical methods for a few protein–ligand systems. One of the most studied macromolecular systems has been the engineered binding pocket of T4 lysozyme. Mobley *et al.* studied the binding of thirteen single-ring fragment-like ligands to a L99A hydrophobic T4 lysozyme cavity mutant, obtaining a root mean square (RMS) error compared to isothermal titration calorimetry (ITC) experiments of roughly 1.9 kcal mol^–1^.^18^ Boyce *et al.* studied instead the binding of similar fragment-like ligands to a slightly polar model cavity of T4 lysozyme (L99A/M102Q) in a prospective fashion, obtaining a RMS error compared to ITC for the five compounds with measurable affinities of about 1.8 kcal mol^–1^.^19^ Another popular test system has been the FK506-binding protein (FKBP12). The series of ligands evaluated with FKBP12 were originally studied experimentally by Holt *et al.* and are drug-like, with multiple rings and several rotatable bonds, although sharing very similar chemical moieties.^20^ Shirts first reported a RMS error of about 2.0 kcal mol^–1^ for the affinity prediction of nine inhibitors,^21^ and a following study by Wang *et al.* obtained an error of 2.0–2.5 kcal mol^–1^.^22^ For this system the experimental free energies taken as reference were derived from competitive inhibition of FKPB12 activity.^20^ Fujitani and coworkers obtained for eight FKBP-12 inhibitors a RMS difference from a linear fit of only 0.4 kcal mol^–1^, however, there was a large offset (–3.2 kcal mol^–1^) relative to experiment.^23^ Other calculations have also been reported albeit on smaller numbers of ligands and this makes it harder to establish the actual errors.^24^–^27^

**Fig. 1:**
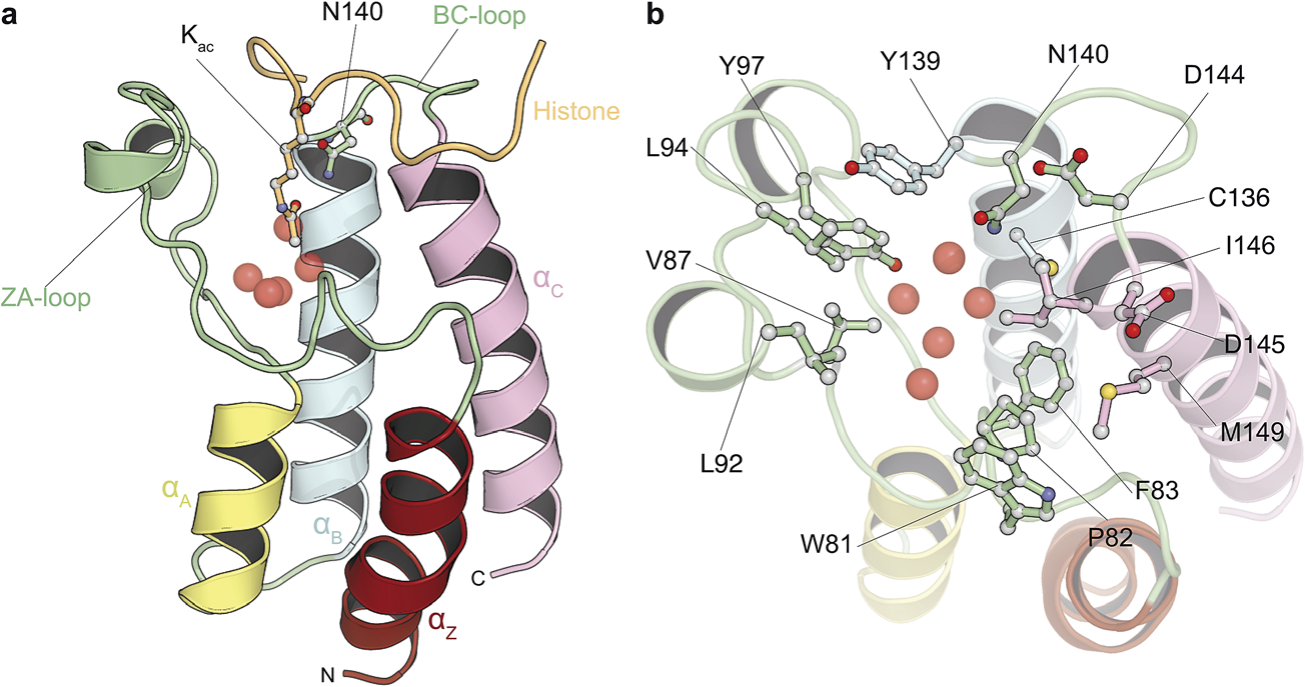
Bromodomain fold and acetyl-lysine binding pocket. (a) Cartoon representation of the structure of BRD4(1) bromodomain in complex with an acetylated peptide. Crystallographically observed water molecules are represented as red spheres. (b) BRD4(1) acetyl-lysine binding site with key interacting residues labeled.

Driven by an interest to support the development of chemical tools *via* simulation methods, we sought to assess whether absolute free energy calculations based on standard implementations of alchemical transformations are now reaching the point where they can be applied to diverse, drug-like organic molecules and pharmacologically relevant targets. In order to achieve our goal, we therefore compared predicted binding free energies for 11 diverse, small molecule inhibitors that bind to bromodomains (BRDs) with experimental measurements, primarily isothermal titration calorimetry (ITC). BRDs are epigenetic mark readers that specifically recognize *ε-N*-lysine acetylation motifs (Fig. 1) and have been found in 46 human nuclear and cytoplasmic proteins.^28^,^29^ Acetylation is often found in macromolecular complexes implicated in chromatin remodeling, DNA repair and cell-cycle control, and especially on histones.^30^ Histone acetylation is thought to result in transcriptional activation and altered acetylation levels have been linked to aberrant transcription in cancer and inflammation.^28^,^31^,^32^ Thus novel BRD inhibitors are finding broad application in medicine and basic biological research^30^ and indeed various BRD inhibitors are currently in phase I and II clinical trial for the treatment of NUT midline carcinoma, acute leukaemia, progressive lymphoma and atherosclerosis.^29^

Here we perform a retrospective analysis in order to assess the performance of the calculations in terms of accuracy and precision in a best-case scenario. We subsequently carry out a pseudo-prospective study where we repeat the exercise with traditional docking methods to give the initial poses without using any structural information for the protein–ligand complexes. Both studies give excellent agreement with experimental data. We discuss the results in terms of how such calculations could be used to aid the drug discovery and development process.

## Methods

### System setup

The initial conformations of the complexes were taken from holo crystal structures (3U5J, ; 3U5L, ; 4OGI, ; 4OGJ, ; 3MXF, ; 4MR3, ; 4MR4, ; 3SVG, ; 4J0R, ; 4HBV) with the exception of ligand 11, which was modeled based on the structure ; 3SVG, and from the results of the ligand docking into the apo protein (; 2OSS). Missing atoms in the crystals were modeled with the WHAT-IF web interface^33^ and all organic molecules that were not the ligand of interest were removed from the system, whereas all crystallographic waters were retained. After adding hydrogens with Maestro (v9.5, Schrödinger), ligands were parameterized with the general AMBER force field (GAFF v1.5)^34^ and AM1-BCC charges^35^ using AmberTools12 36 provided with the FESetup tool v1.1pre1 (; http://ccpforge.cse.rl.ac.uk/gf/project/ccpbiosim). GROMACS topologies and coordinates were generated from the AMBER ones using acpype (v.2013-11-28 Rev: 399).^37^ We used the Amber99SB-ILDN force field^38^ for the protein and the TIP3P model^39^ for water molecules. The complexes were solvated in a dodecahedral box, apart from ligands 1 and 4 that were solvated in a cubic box, with periodic boundary conditions and a minimum distance between the solute and the box of 12 Å. Sodium and chloride ions were added to neutralize the systems.

### Free energy calculations

**Fig. 2:**
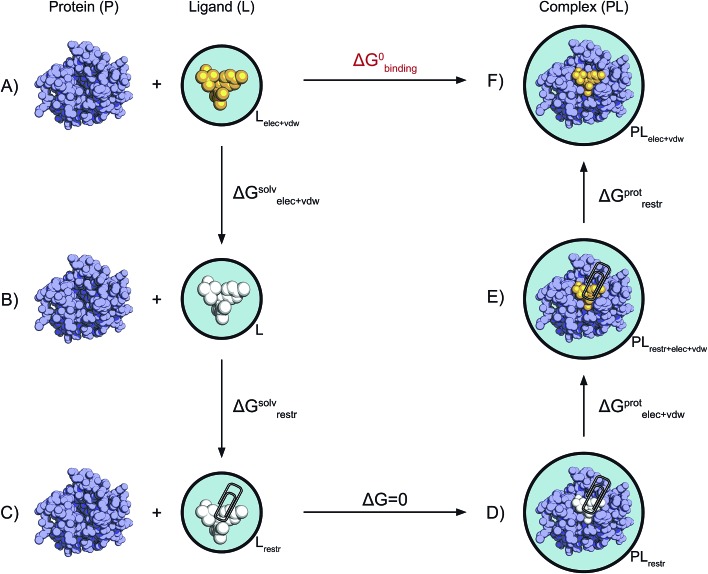
Non-physical thermodynamic cycle. Scheme of the alchemical thermodynamic cycle used to obtain the absolute binding free energies. The fully interacting ligand (orange) in solution at the top left (A) is transformed into a non-interacting solute (B, white) during a series of equilibrium simulations where its electrostatic and van der Waals interactions are scaled to zero, providing the term Δ*G*solvelec+*v*d*w*. The ligand is then restrained while still non-interacting with the environment (C). This step (Δ*G*solvrestr) is computed analytically in accordance with the protocol described by Boresch *et al.*^47^ This state is equivalent to having the non-interacting ligand restrained within the protein cavity (D). The restrained and non-interacting ligand in complex with the protein has its electrostatic and *V*d*W* interactions turned back on again (E), giving Δ*G*protelec+*v*d*w*. The restraints between ligand and protein are then removed (Δ*G*protrestr), closing the cycle, and the final state is the unrestrained and fully interacting ligand in complex with the protein (F).

Absolute binding free energy calculations were performed beginning from both crystal ligand poses and docked poses as detailed in the Results section, using the non-physical thermodynamic cycle illustrated in Fig. 2. All simulations were carried out in GROMACS 4.6.5.^40^,^41^ The ligand van der Waals interactions were decoupled and the charges annihilated using a linear alchemical pathway with Δ*λ* = 0.05 for the van der Waals and Δ*λ* = 0.1 for the coulombic transformations. For the addition of the ligand restraints instead, 12 non-uniformly distributed *λ* values were used (0.0, 0.01, 0.025, 0.05, 0.075, 0.1, 0.15, 0.2, 0.3, 0.5, 0.75, 1.0). A total of 42 windows for the complex simulations and 31 windows for the ligand simulations were therefore employed. For each window, 10 000 energy minimization steps were carried out using a steepest descent algorithm. The system was subsequently simulated for 0.5 ns in the canonical ensemble with harmonic position restraints applied to the solute heavy atoms with a force constant of 1000 kJ mol^–1^ nm^–2^. Temperature was coupled using Langevin dynamics^42^,^43^ with 298.15 K as the reference temperature. A 1 ns position restrained run in the isothermal–isobaric ensemble was then performed using the Berendsen weak coupling algorithm.^44^ 10 ns unrestrained production runs were performed for data collection using Hamiltonian-exchange Langevin dynamics with a 2 fs time-step in the NPT ensemble with the Parrinello–Rahman pressure coupling scheme.^45^ 3 million swaps between any state pair were attempted every 1000 time steps, following the Gibbs sampling scheme proposed by Chodera & Shirts.^46^ This led to acceptance rates between neighbouring states that ranged from 0.1 to 0.3 (mean and standard deviation of 0.2 ± 0.1), but a probability of jumping to any other state from 0.2 to 0.9 (mean and standard deviation of 0.7 ± 0.2). This resulted in a total unrestrained simulated time of 43 μs for this study. The relative position and orientation of the bound ligand with respect to the protein was restrained by means of one distance, two angles and three dihedral harmonic potentials with force constant of 10 kcal mol^–1^ Å^–2^ [deg^–2^]. The contribution of this set of restraints to the free energy can be calculated analytically as described by Boresch *et al.*^47^ for the non-interacting ligand in solution (Δ*G*solvrestr), while it has to be evaluated numerically for the interacting ligand in complex with the protein (Δ*G*protrestr). The equation used to evaluate this contribution also includes a correction for the standard-state dependence of the binding free energy.^47^ A soft-core potential^48^ was employed for the van der Waals interactions transformed. For all simulations the particle mesh Ewald (PME) algorithm^49^ was used for electrostatic interactions with a real space cut-off of 12 Å, a spline order of 6, a relative tolerance of 10^–6^ and a Fourier spacing of 1.0 Å. A switch function between 9 Å and 10 Å was used for the van der Waals interactions. The P-LINCS constraint algorithm^50^ was used only on H-bonds. The GROMACS long-range dispersion correction for energy and pressure was used, and an additional long-range dispersion correction (EXP-LR) was applied as described in Shirts *et al.*^51^ For charged compounds (1 and 4), the semi-analytical correction scheme for electrostatic finite size effects proposed by Rocklin *et al.*^52^ was employed. The residual integrated potential (RIP) was calculated for 11 frames (every 1 ns, from 1 ns to 10 ns) using APBS 1.3.^53^ The mean RIP value only was used for the correction; its standard deviation, resulting from the different conformations of the ligand and complex, was always below 0.05 kcal mol^–1^.

### Data analysis

The results were analysed with the implementation of the multiple Bennet acceptance ratio (MBAR) provided with the python package pymbar (https://simtk.org/home/pymbar).^54^ The first 1 ns of each window was discarded as an equilibration period. Prior to the free energy estimate, the data from each lambda state were subsampled in order to include only uncorrelated data-points by calculating the autocorrelation time and statistical inefficiency of the potential energy. 200 boot-strap sets were constructed by random resampling with replacement of the uncorrelated data, where the first set was the original sample. The free energy was estimated with MBAR for all of the 200 sets and the final estimate is the mean of all these free energy estimates.^55^ The error of the final estimate is the sample standard deviation of the estimates for all bootstrap samples. For the calculations beginning from the crystal ligand poses, the whole calculations have been repeated three times in order to assess the convergence of the results. The combined free energy estimate and standard deviation for ligand and complex simulations were determined by taking the mean and sample standard deviation of all the 600 bootstrap samples. Therefore, the final uncertainty is representative of both the statistical uncertainty of the MBAR free energy estimate and the error due to finite sampling. Ligand 11 is an exception, as it was modeled based on ligand 9 and the PDB structure ; 3SVG, and two equally plausible binding modes were present, where the trifluorotoluene moiety is flipped by 180°. Therefore, two calculation repeats were carried out per binding mode, resulting in a total of four binding free-energy calculations; the results of multiple binding modes can be combined in a single binding free-energy value as described by Mobley *et al.*^56^ The final binding free energy for each ligand is the difference between the decoupling of the ligand from the water solution and from the solvated complex; the final error in the binding free energies is thus the root sum square of the uncertainties of ligand and complex calculations.

### Docking

Rigid docking was performed with MOE v2013.08 using a crystal structure (PDB 2OSS) of the apo protein of BRD4(1). The five highly conserved crystal waters present in all BET bromodomains binding pockets were kept, whereas all other waters and organic molecules were removed. The ligands' 2D chemical structures were drawn in Marvin Sketch (v6.1.0, ChemAxon) and a stochastic conformational search was performed in order to generate 3D conformations. The number of conformations was limited to a maximum of 100 per ligand and duplicates conformations (RMSD < 0.25 Å) were removed. The docking protocol employed the pharmacophore placement method and the London Δ*G* scoring function. Each binding pose was then minimized and rescored with the GBVI/WSA Δ*G* scoring function. The pharmacophore query was built based on the properties of the acetyl-lysine found in the PDB structure ; 3UVW, and consisted of a hydrogen-bond acceptor site, to mimic the acetyl oxygen, and a non-polar site, corresponding to the position of the methyl moiety. The protein was parameterized using Amber ff99SB.^57^ The ligand bonded parameters were obtained with 2D extended Hückel theory,^58^ the *V*d*W* parameters were derived from GAFF^34^ and the charges from Bond Charge Increments^59^ according to the AMBER10:EHT force field option in MOE. Duplicate poses were automatically removed based on their hydrogen-bond and hydrophobic patterns, and poses with positive binding free energy as predicted by the GBVI/WSA Δ*G* scoring function were removed too, as they typically involved clear clashes with the protein atoms. The remaining poses were furthermore clustered by RMSD with a 3 Å cut-off in order to have a coarser landscape of the possible binding poses, also considering we were not interested in running the free energy calculations on similar binding orientation that can interconvert within the simulations timescale. Thus, only the best scoring pose within each cluster would be used for free energy calculations. This procedure aimed at reducing the number of calculations to run while maximizing the chances of retaining the poses that closely approximate the crystal.

## Results

### Absolute free energy calculations based on crystal structures are accurate and precise

**Fig. 3:**
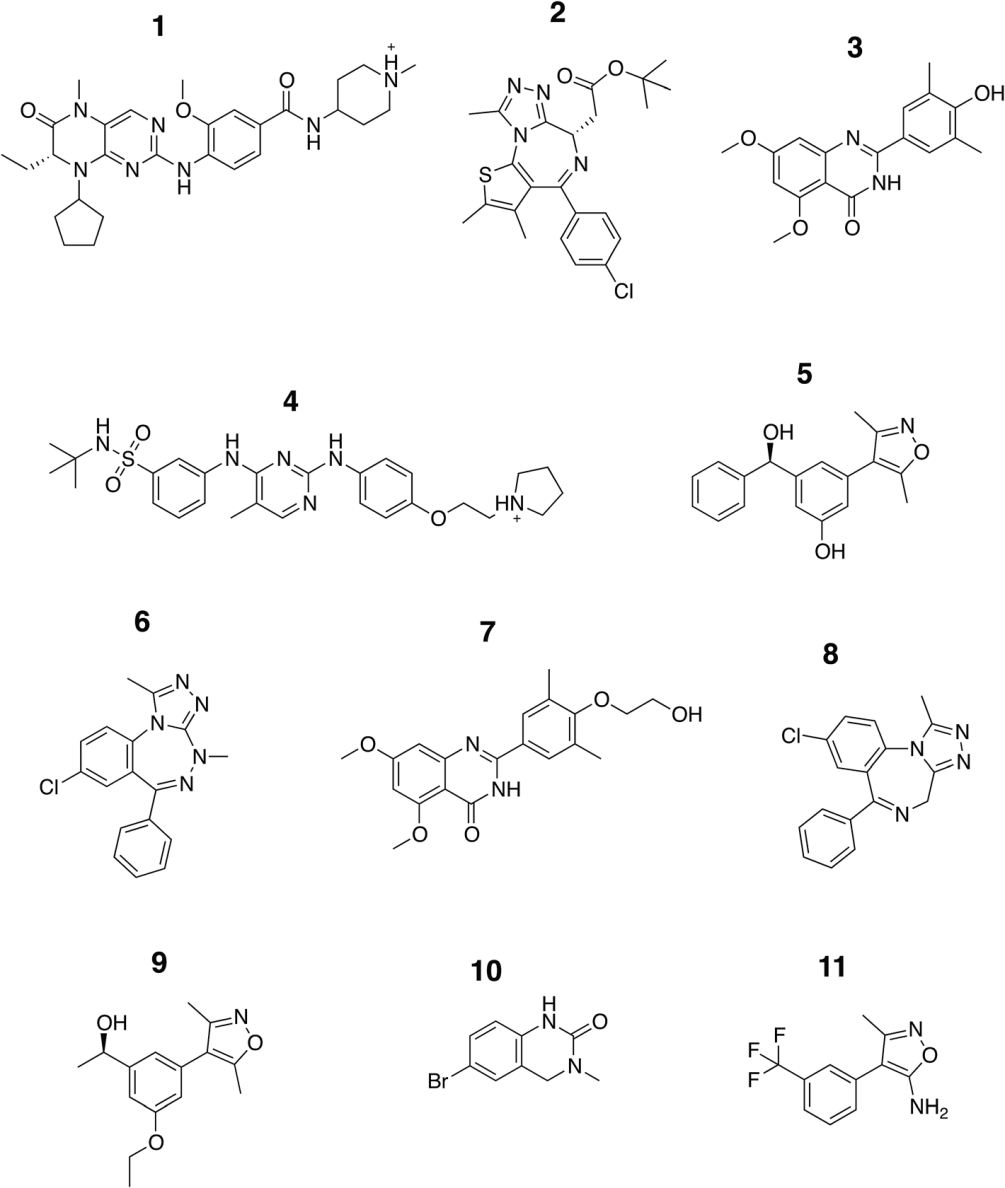
Chemical structure of the ligands. The structures of the compounds analyzed in this study are shown and are labeled with Arabic numerals in descending order of affinity.

In this study we carried out thorough binding free energy calculations using a non-physical thermodynamic cycle (Fig. 2), starting from the crystal structures of BRD4(1) in complex with 11 inhibitors to a common binding site (Fig. 1, ESI Fig. 1†). We were first interested in evaluating the performance of the predictions in a favorable scenario, that is, when the binding conformation is known from experiment. The results from this study, therefore, provide a picture of the best accuracy that can be expected. In addition, we were interested in evaluating the precision of the calculations, considering that large and flexible molecules are present in the test set (Fig. 3). Large uncertainties in the results when dealing with such drug-like molecules would indeed prevent a meaningful assessment of the accuracy of the results. To this end, in addition to the bootstrap analysis to evaluate the statistical uncertainty of the free energy estimator, we decided to repeat the calculations three times, in order to obtain an approximation of the uncertainty due to finite sampling (for ligand 11, four repeats were performed, as explained in the Methods section). It was in fact noticed that while bootstrap provided a more realistic uncertainty estimate than the MBAR error estimate alone, it still underestimated the sample standard deviation. Each calculation was the results of 73, 10 ns long, all-atom molecular dynamics runs for a total simulated time of about 25 μs for this portion of the study.

The set of inhibitors considered comprises mostly drug-like molecules with a diverse range of physicochemical properties: number of atoms from 22 to 77; molecular weight from 241 to 525 Da; number of rotatable bonds from 0 to 11; calculated log *P* from –0.4 to 5.3 (ESI Table 1†). The range of affinities includes micromolar binders such as ligand 10 (∼23 μM) and 11 (∼80 μM), down to low nanomolar binders such as ligand 1 (∼40 nM) and 2 (∼50 nM). A number of different chemical groups are represented and the dissimilarity of the set provides us with more confidence that the results obtained are not excessively biased by the limited chemical space considered.

Table 1 summarizes the results obtained for this retrospective study (see ESI Table 2† for a breakdown of the energetic contributions). Most calculations agree extremely well with the experimentally determined values. Seven out of eleven predictions have errors below 0.5 kcal mol^–1^, and all prediction errors are below 2.0 kcal mol^–1^. This resulted in a mean absolute error (MAE) of 0.6 ± 0.1 kcal mol^–1^ and a root mean square (RMS) error of 0.8 ± 0.2 kcal mol^–1^. The calculated free energies strongly correlate with the experimental ones, as shown in Table 1 and Fig. 5, with a Pearson's *r* of 0.84 ± 0.05, and manage to rank the ligand affinities effectively (Spearman's *ρ* = 0.82 ± 0.06). The precision of the calculations is encouraging too, as in only three instances the uncertainty is above 0.5 kcal mol^–1^, and in all case it is below 1.0 kcal mol^–1^ (see ESI Fig. 2† for convergence assessment). The largest uncertainties, as expected, occur when the largest ligands are considered.

**Table 1:** Summary of free energy calculation results based on crystal structures. Δ*G*_calc_ is the calculated standard binding free energy; Δ*G*_exp_ is the experimental standard binding free energy. Reported are also the PDB files used as input and the experimental method used for the affinity measurement. All values are in kcal mol^–1 α^. All errors are one standard deviation. This is an estimate of the typical ITC standard deviation (1*σ*) based on the variability of the affinity values observed in the ABRF-MIRG′02 inter-laboratory assessment;^67^^β^the error represents the standard deviation of two measurements; ^γ^no error reported as only a single experiment was performed. Values for the difference between Δ*G*_calc_ and Δ*G*_exp_ might appear inconsistent due to rounding

Cpd	Δ*G*_calc_	Δ*G*_exp_	Δ*G*_calc_–Δ*G*_exp_	PDB	Exp method	Reference
1	–10.4 ± 0.6	–9.8 ± 0.1^α^	–0.6 ± 0.6	4OGI	ITC	68
2	–9.5 ± 0.4	–9.6 ± 0.1^α^	+0.2 ± 0.4	3MXF	ITC	69
3	–9.2 ± 0.5	–9.0 ± 0.1^α^	–0.2 ± 0.5	4MR3	ITC	70
4	–9.4 ± 0.8	–8.9 ± 0.1^α^	–0.4 ± 0.8	4OGJ	ITC	68
5	–8.6 ± 0.3	–8.8 ± 0.1^β^	+0.2 ± 0.3	4J0R	SPR	71
6	–9.9 ± 0.8	–8.2 ± 0.1^α^	–1.7 ± 0.8	3U5L	ITC	72
7	–5.9 ± 0.5	–7.8 ± 0.1^α^	+2.0 ± 0.5	4MR4	ITC	70
8	–7.8 ± 0.3	–7.4 ± 0.1^α^	–0.4 ± 0.3	3U5J	ITC	72
9	–7.7 ± 0.4	–7.3 ± 0.0^β^	–0.4 ± 0.4	3SVG	AlphaScreen	71
10	–5.9 ± 0.2	–6.3 ± 0.1^β^	+0.4 ± 0.3	4HBV	AlphaScreen	73
11	–5.4 ± 0.2	–5.6^γ^	+0.1 ± 0.2	Model	AlphaScreen	74

*Statistics*	
Mean absolute error	0.6 ± 0.1
Root mean square error	0.8 ± 0.2
Pearson's *r*	0.84 ± 0.05
Spearman's *ρ*	0.82 ± 0.06

### Accurate predictions can also be achieved in absence of structural information about the complex

**Fig. 4:**
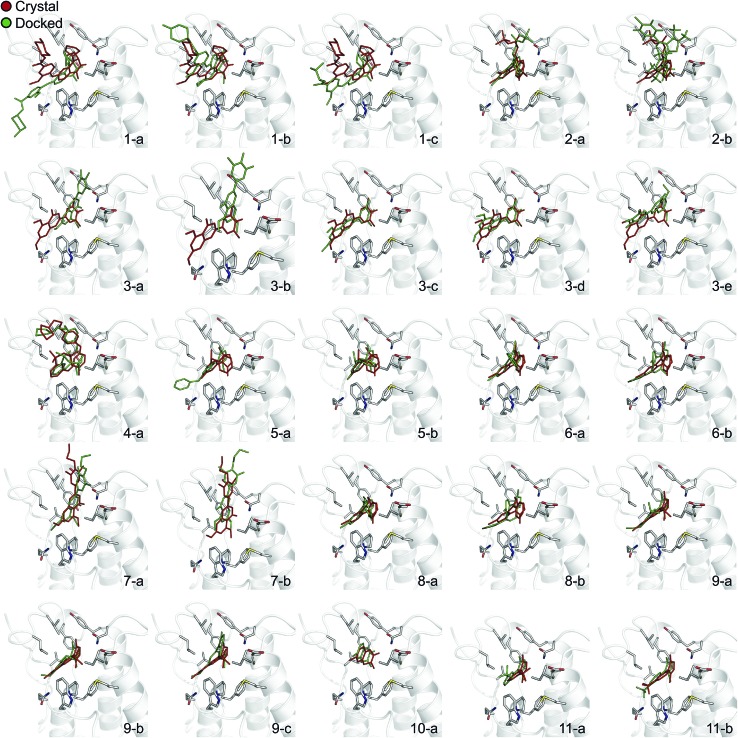
Binding poses suggested by docking. In red are the crystallographic structures, and in green are the docked ligands. The ligand number and cluster letter are reported on each pose.

In order to evaluate the usefulness of such calculations in a prospective context, we carried out a docking and free energy calculation exercise based upon docking results rather than crystal structures. The main objective of this portion of the study was to evaluate whether the accuracy observed in the retrospective exercise could be achieved with a more prospective docking-based approach. Three-dimensional ligand structures were therefore generated through a conformational search after drawing the ligands in two dimensions. A pharmacophore based on acetyl-lysine was then created and used to aid the docking of the 11 inhibitors to an apo structure of BRD4(1) (pdb-ID 2OSS). Unrestrained molecular dynamics simulations explore binding conformations close to each other, hence the docking poses obtained were clustered by root mean square deviation (RMSD) in order to avoid selecting conformations that interconvert during the simulations time scales. The total number of docked poses obtained was 72, which was reduced to 25 after the clustering procedure and removal of poses with positive binding free energies as predicted by the scoring function. Fig. 4 shows these 25 docking poses (scores and RMSD to crystal are summarized in ESI Table 3†).

**Fig. 5:**
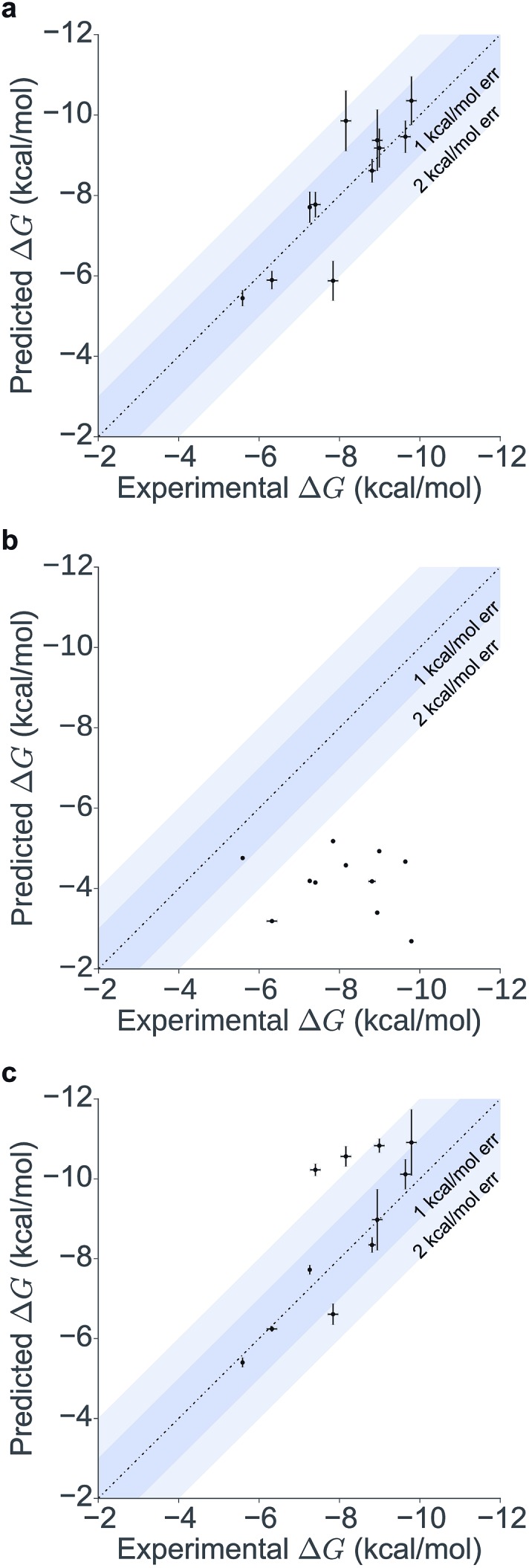
Scatter and correlation plots of the results. Correlation plots for (a) the free energy calculations starting from the X-ray structures, (b) the docking free energy scores and (c) the free energy calculations starting from the docked structures.

For all ligands, a binding pose that captures the main features of its interactions with BRD4(1) was among the results. The docking software managed to reproduce the binding mode of the inhibitors well. For eight out of ten ligands the RMSD is below 2.0 Å. In addition, docking correctly identified the pose closest to the crystallographic observed one as being the most favorable for 7 out of 10 ligands. For ligand 1, two poses actually capture the correct binding mode of the molecules within BRD4(1) binding pocket (poses 1-a and 1-c), despite the fact pose 1-a appears to be substantially different from the crystal (RMSD of 8.4 Å). This is however due to the fact that a large part of this inhibitor is solvent exposed and thus free to explore a number of conformations. The extensive sampling in the unrestrained simulations means that such deviation from the crystal structure does not affect the free energy result (while it does affect the docking score), and the ligand is still predicted to be a strong binder when starting from pose 1-a (ESI Table 4†). Docking, despite providing good binding conformations for the ligands, and fairly good relative pose ranking for the same ligand, scored the ligands inaccurately (Fig. 5b). With a RMS error of 4.2 kcal mol^–1^ and a Pearson's *r* of –0.16, the affinities provided do not help in discriminating between tight and weak binders. Conversely, the free energy calculations based on MD still managed to have excellent agreement with the experimental affinities (Fig. 5c). Table 2 (full breakdown in ESI Table 4†) reports the results of the free energy calculations based on the lowest energy docked poses, along with the RMSD of the poses with respect to the crystal ones and the binding free energy predicted by the docking scoring function for the same ligand. Mean absolute and root mean squared errors were respectively 1.0 ± 0.1 kcal mol^–1^ and 1.4 ± 0.1 kcal mol^–1^, whereas the correlation to experimental values was of 0.77 ± 0.04 for Pearson's *r* and of 0.72 ± 0.08 for Spearman's *ρ*.

**Table 2:** Summary of the free energy calculation results based on docking. Shown are the data for the predicted most stable binding poses for each ligand. Δ*G*_calc_ is the calculated standard binding free energy; Δ*G*_exp_ is the experimental standard binding free energy. For comparison, also the affinities predicted with the docking scoring function are reported. All values are in kcal mol^–1^. All errors are one standard deviation. “X-ray pose” indicates whether the lowest energy pose identified corresponds to the crystallographically observed binding mode; also the RMSD of the pose as compared to the crystal is reported. ^α^This is an estimate of the typical ITC standard deviation (1*σ*) based on the variability of the affinity values observed in the ABRF-MIRG′02 inter-laboratory assessment;^67^^β^the error represents the standard deviation of two measurements; ^γ^no error reported as only a single experiment was performed. Difference values may include rounding effects

Compound	Δ*G*_calc_	Δ*G*_exp_	Δ*G*_calc_–Δ*G*_exp_	X-ray pose	RMSD (Å)	Docking Δ*G*
1	–10.9 ± 0.8	–9.8 ± 0.1^α^	–1.1 ± 0.8	Yes	3.2	–2.7
2	–10.1 ± 0.4	–9.6 ± 0.1^α^	–0.5 ± 0.4	Yes	1.9	–4.7
3	–10.8 ± 0.2	–9.0 ± 0.1^α^	–1.8 ± 0.2	Yes	2.0	–4.9
4	–9.0 ± 0.8	–8.9 ± 0.1^α^	–0.0 ± 0.8	Yes	1.8	–3.4
5	–8.3 ± 0.2	–8.8 ± 0.1^β^	+0.5 ± 0.2	Yes	1.2	–4.2
6	–10.6 ± 0.3	–8.2 ± 0.1^α^	–2.4 ± 0.3	No	5.0	–4.6
7	–6.6 ± 0.3	–7.8 ± 0.1^α^	+1.2 ± 0.3	Yes	2.2	–5.2
8	–10.2 ± 0.2	–7.4 ± 0.1^α^	–2.8 ± 0.2	Yes	0.8	–4.2
9	–7.7 ± 0.1	–7.3 ± 0.0^β^	–0.5 ± 0.1	Yes	1.8	–4.2
10	–6.2 ± 0.1	–6.3 ± 0.1^β^	+0.1 ± 0.2	Yes	0.7	–3.2
11	–5.4 ± 0.1	–5.6^γ^	+0.2 ± 0.1	n.a.	n.a.	–4.8

*Statistics*	
Mean absolute error	1.0 ± 0.1
Root mean square error	1.4 ± 0.1
Pearson's *r*	0.77 ± 0.04
Spearman's *ρ*	0.72 ± 0.08

### Absolute calculations can resolve ambiguities between multiple potential binding modes

**Fig. 6:**
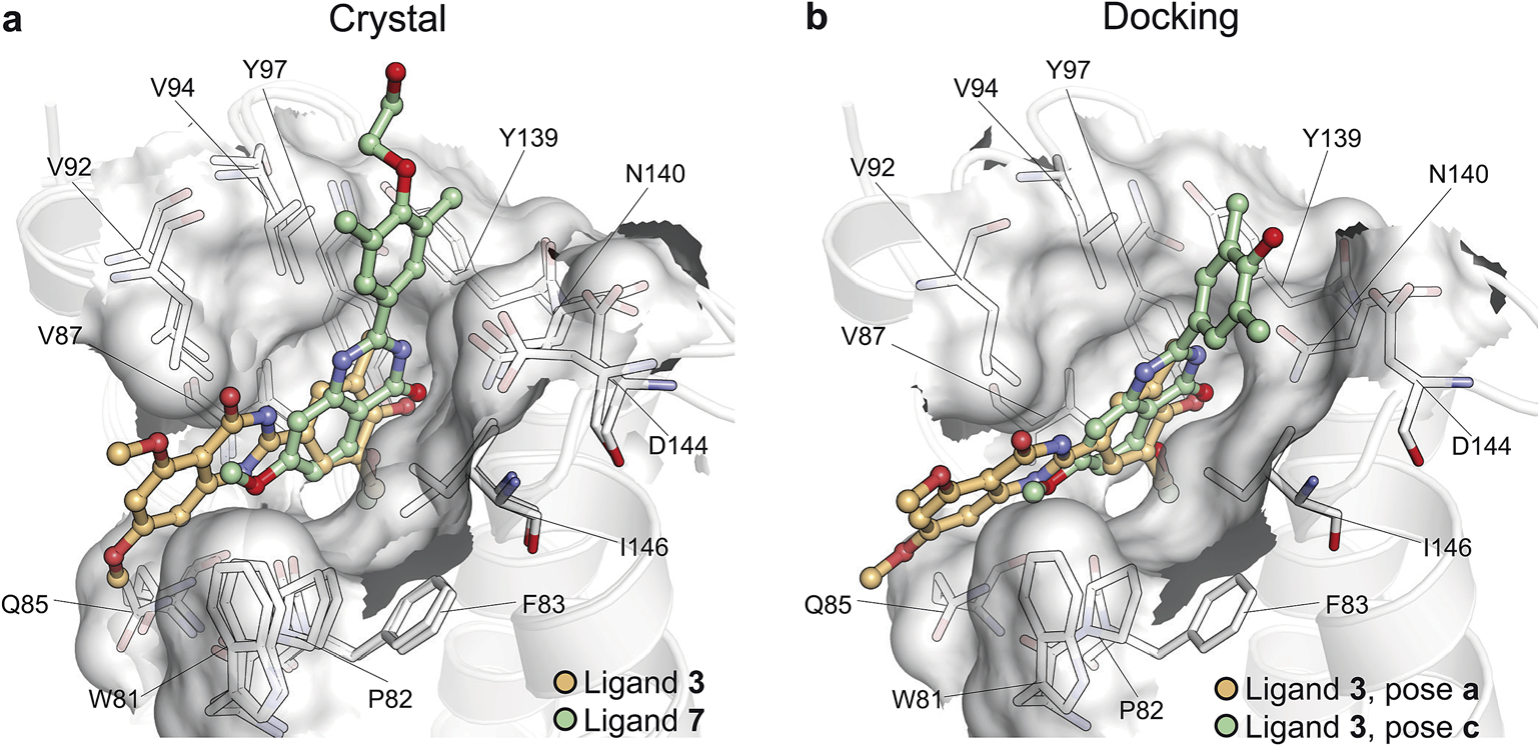
Multiple potential binding conformations of ligand 3. (a) Overlay of the crystal structures for ligand 3 and ligand 7 (4MR3 and ; 4MR4 respectively). (b) Overlay of docking poses 3-a and 3-c.

To illustrate the potential of alchemical calculations in resolving ambiguous binding poses, the case of ligand 3 is presented in more detail. Ligands 3 and 7 are closely related; in fact ligand 3 is the synthetic precursor of ligand 7. Despite their chemical similarity, the two ligands bind the BRD4(1) binding pocket in two very different modes, as shown in Fig. 6a. This substantial change in binding pose is extremely hard to predict by visual inspection or docking alone. Indeed, the most favorable binding pose (pose 3-a, docking score of –4.9 kcal mol^–1^) for ligand 3 proposed by docking closely resembled the pose of ligand 7 (Fig. 6b), which forms two hydrogen bonds with N140 through the dihydroquinazolinone scaffold and buries a methoxy group at the bottom of the pocket. Pose 3-b was assigned the second best docking score (–3.5 kcal mol^–1^) and occupied the same cleft as pose 3-a, however, with the amide that is part of the dihydroquinazolinone scaffold pointing away from N140, the double hydrogen bond to it is lost. These poses thus have a large RMSD as compared to the X-ray pose (6.8 Å and 7.8 Å for poses 3-a and 3-b respectively). The actual binding mode of the ligand is correctly represented instead by the pose 3-c, which is assigned a worse docking score (–2.6 kcal mol^–1^) than 3-a and 3-b, and it is characterized by the formation of one hydrogen bond with N140 thanks to the oxygen of the dimethylphenol ring, and the burial of a methyl group in the hydrophobic pocket in an analogous fashion to the binding of the acetyl moiety in Kac. Pose 3-d binds BRD4(1) through a similar pose as 3-c, forming one hydrogen bond with N140 through the hydroxyl group and burying a methyl group deeply in the protein binding pocket. However, while in 3-c and in the X-ray structure the amide moiety of the dihydroquinazolinone group points towards the solvent, in 3-d this is directed toward the protein. As a consequence, pose 3-d shows an RMSD as compared to the X-ray pose that is slightly larger (3.0 Å) than for pose 3-a (2.0 Å). Pose 3-e occupies a similar volume to 3-c and 3-d, but the dimethylphenol group responsible for binding is solvent exposed and the two methoxy groups are instead directed toward N140, resulting in a pose that overall has few contacts with the protein and is very far from the crystal pose as suggested by the large RSMD (7.8 Å).

Absolute free energy calculations were carried out starting from all docking structures in order to evaluate whether the method could unambiguously determine the lowest energy pose in this challenging case. The binding free energy obtained for the pose that best approximates the bound structure in the crystal (pose 3-c), was –10.8 ± 0.2 kcal mol^–1^, whereas the free energy for the pose that binds BRD4(1) similarly to ligand 7 (pose 3-a) was estimated to be –6.2 ± 0.2 kcal mol^–1^. Pose 3-d, which is the second closest to the X-ray structure and retains the main interaction patters, was estimated to have a high binding affinity too (–10.5 ± 0.2 kcal mol^–1^). On the other hand, poses 3-b and 3-e were predicted to have significantly lower binding affinities (–6.5 ± 0.3 kcal mol^–1^ and –7.3 ± 0.2 kcal mol^–1^ respectively) than 3-c. The results therefore unequivocally identified the crystallographic binding pose as being the most favorable one.

There is only one case where the free energy calculations appear to be unable to unambiguously identify the most stable binding pose and that is ligand 6. In this case both the scoring function and the MD suggest that the two poses (6-a and 6-b) have similar binding affinity for BRD4(1). Interestingly, ligand 6, when compared to the similar ligand 8, has an additional methyl group on its triazepine ring that can potentially mimic the methyl moiety of the acetylated lysine. Indeed, pose 6-b binds the pocket placing such methyl group similarly to Kac. Pose 6-b might therefore be a legitimate secondary binding pose, even though its binding affinity is likely overestimated.

## Discussion

As discussed by Mobley and Klimovitch,^60^ reliable binding free energy predictions can have a substantial impact in drug discovery campaigns even with modest levels of accuracy. In a lead optimization exercise, screening ∼10–100 molecules per week with 2.0 kcal mol^–1^ of noise would reduce the synthetic effort by a factor of 3 when the goal is to achieve a 10-fold improvement in binding affinity (*i.e.* a 1.4 kcal mol^–1^ improvement in binding free energy). Moreover, absolute calculations need only structural information of the target in order to be employed. Despite currently still being computationally expensive, at this level of accuracy it is easy to recognize the great potential for application in lead optimization campaigns in a near future, complementing relative calculations.^61^ Assuming steady improvements in hardware and algorithmic performance, in the long term it is possible to foresee applications in lead discovery too as an accurate rescoring method. Furthermore, we showed how alchemical calculations are able to resolve ambiguities regarding unexpectedly large differences in binding modes between extremely similar molecules. The precision of the calculations was rigorously assessed in order to take into account both the statistical and sampling uncertainties. We have shown how for even the largest and most flexible ligands standard deviations below 1.0 kcal mol^–1^ are achievable within the microsecond time-scale. It is important however to remember that the accuracy of such calculations comes at a high computational cost with respect to scoring functions or endpoint methods. For each calculation, the production simulations for the complex took on average ∼29 hours on 504 cores (Intel Xeon E5-2697 v2 2.7 GHz), and ∼7 hours on 372 cores for the ligand. While the use of graphical processing units can substantially accelerate the simulations, the screening of hundreds to thousands of compounds would still be a very onerous exercise. Nonetheless, the accurate experimental determination of binding affinities using biophysical methods such as ITC can be very time consuming too. Before the calorimetric experiment can be prepared and executed, protein expression and purification alone can take the order of a week, and the ligand of interest might need to be synthesized through multiple reaction steps. In addition, typically large quantities of titrant are needed to carry out the experiment.

An interesting feature of absolute calculations is the possibility to account for multiple binding poses in the final binding free energy estimate. This has been shown to be important to accurately estimate the affinity of small fragments,^18^ which by having a limited number of interactions with the protein, and less shape complementarity requirements as compared to drug-like molecules, may adopt multiple binding modes with similar thermodynamic stability. Nonetheless, a requirement for successful absolute binding free energies is to have a starting structure that captures the main feature of the protein–ligand complex. We showed how, thanks to the ability of MD to explore an ensemble of conformations, having a starting ligand pose that considerably deviates from the crystal pose does not affect the results as long as the main features of the binding are maintained. However, the methodology is still heavily dependent on the quality of poses generated by docking. An incorrect pose prediction will lead to a false negative when calculating the ligand affinity. In a recent methodological advance it has been shown how it is possible to combine Hamiltonian replica exchange with Monte Carlo ligand translation/rotation moves to simultaneously estimate binding free energies and identify ligand binding sites and orientations.^62^ Further developments in such direction, coupled with increasing computing power, might alleviate the need to rely on faster and less accurate methods such as docking for pose prediction. Nevertheless, in this study, docking was sufficient and succeeded in finding good poses for all of the inhibitors considered, while visibly failing to rank them or estimate their binding energies. The latter is a well-known limitation of scoring functions.^63^ These were however accurately estimated by the MD-based calculations, thus making the docking scores ultimately irrelevant for the final results. The accuracy of free energy calculations is dependent on other force field parameters too. The effect of van der Waals and coulombic non-bonded parameters on binding free energy results has been previously discussed^18^,^64^,^65^ including using QM calculations to handle polarization better.^66^ When dealing with drug-like ligands it is apparent that torsional parameters can also affect the performance of the calculations.^75^,^76^ However, it is encouraging that current MM parameters despite their approximations manage to provide the level of accuracy here presented, which could be even improved by simple refinement and extension of existing models. With small molecules force fields being constantly revised in order to better cover the large chemical space of organic compounds, MD-based affinity predictions hold great promise for the future of structure-based drug design.

## Conclusion

We have shown here that for a small and fairly rigid system such as a bromodomain, free energy calculations based on molecular dynamics are able to achieve RMS errors that do not exceed 1.4 kcal mol^–1^ when starting from docked structures, and down to 0.8 kcal mol^–1^ when using crystal structures and a more expensive protocol. The present results corroborate the potential of absolute free energy calculations for drug discovery applications. To our knowledge, this is the first study on absolute binding free energy that takes into account a diverse set of drug-like molecules and a biologically relevant target currently investigated for its therapeutic potential. Notably, a similar level of accuracy was recently reported for a large set of molecules in terms of their relative binding free energies.^61^ The reliability of the absolute free energy calculations warrants their use in drug discovery campaigns at least for fairly rigid drug targets such as bromodomains.

## Conflict of interest

AH and MJB are employees of Evotec. There are no competing financial interests to declare.

## Supplementary Material

Supplementary information
