# Supplementary material for: Accurate calculation of the absolute free energy of binding for drug molecules
Source: Chem Sci. 2015 Sep 25;7(1):207–18. doi: 10.1039/c5sc02678d (PMC4700411; doi:10.1039/c5sc02678d)
Supplement: Supplementary file 1 [file SC-007-C5SC02678D-s001.pdf]

Electronic Supplementary Material (ESI) for Chemical Science.
This journal is © The Royal Society of Chemistry 2015

Accurate Calculation of the Absolute Free Energy of Binding for Drug Molecules

Supplementary Information

Matteo Aldeghi[1], Alexander Heifetz[2], Michael J. Bodkin[2], Stefan Knapp[3,4] and Philip C. Biggin[1]*

[1]Structural Bioinformatics and Computational Biochemistry, Department of Biochemistry, University of Oxford, South Parks Road, Oxford, OX1 3QU, UK.

[2]Evotec (U.K.) Ltd., 114 Innovation Drive, Milton Park, Abingdon, Oxfordshire OX14 4RZ, U.K.

[3]Structural Genomics Consortium, Nuffield Department of Clinical Medicine, University of Oxford, Old Road Campus Research Building, Roosevelt Drive, Oxford OX3 7DQ, UK.

[4]Target Discovery Institute, Nuffield Department of Clinical Medicine, University of Oxford, Roosevelt Drive, Oxford OX3 7BN, UK.

*To whom correspondence should be addressed.

Email: philip.biggin@bioch.ox.ac.uk

Tel. +44 1865 613305

Fax. +44 1865 613238

Ligand	MW (Da)	No Atoms	Netq (*e*)	NROT	HBA	HBD	cLogP
**1**	522.7	78	+1	8	10	2	-0.4 (3.1)§
**2**	457.0	56	0	5	6	0	5.3
**3**	326.4	42	0	3	6	2	3.0
**4**	525.7	74	+1	11	9	3	1.4 (4.9)§
**5**	295.3	39	0	3	4	2	3.0
**6**	323.8	37	0	1	5	0	3.8
**7**	370.4	49	0	6	7	2	2.5
**8**	308.8	35	0	1	4	0	3.0
**9**	261.3	38	0	4	4	1	2.2
**10**	241.1	22	0	0	3	1	1.8
**11**	242.2	26	0	2	3	2	2.3

Supplementary Table 1: **Physico-chemical properties of the ligands**. MW is the molecular weight in Daltons; Netq is the net charge; NROT is the number of rotatable bonds; HBA is the number of hydrogen bond acceptors; HBD is the number of hydrogen bond donors; cLogP is the calculated octanol/water partition coefficient (computed with MarvinSketch); § cLogP of the nonionic specie.

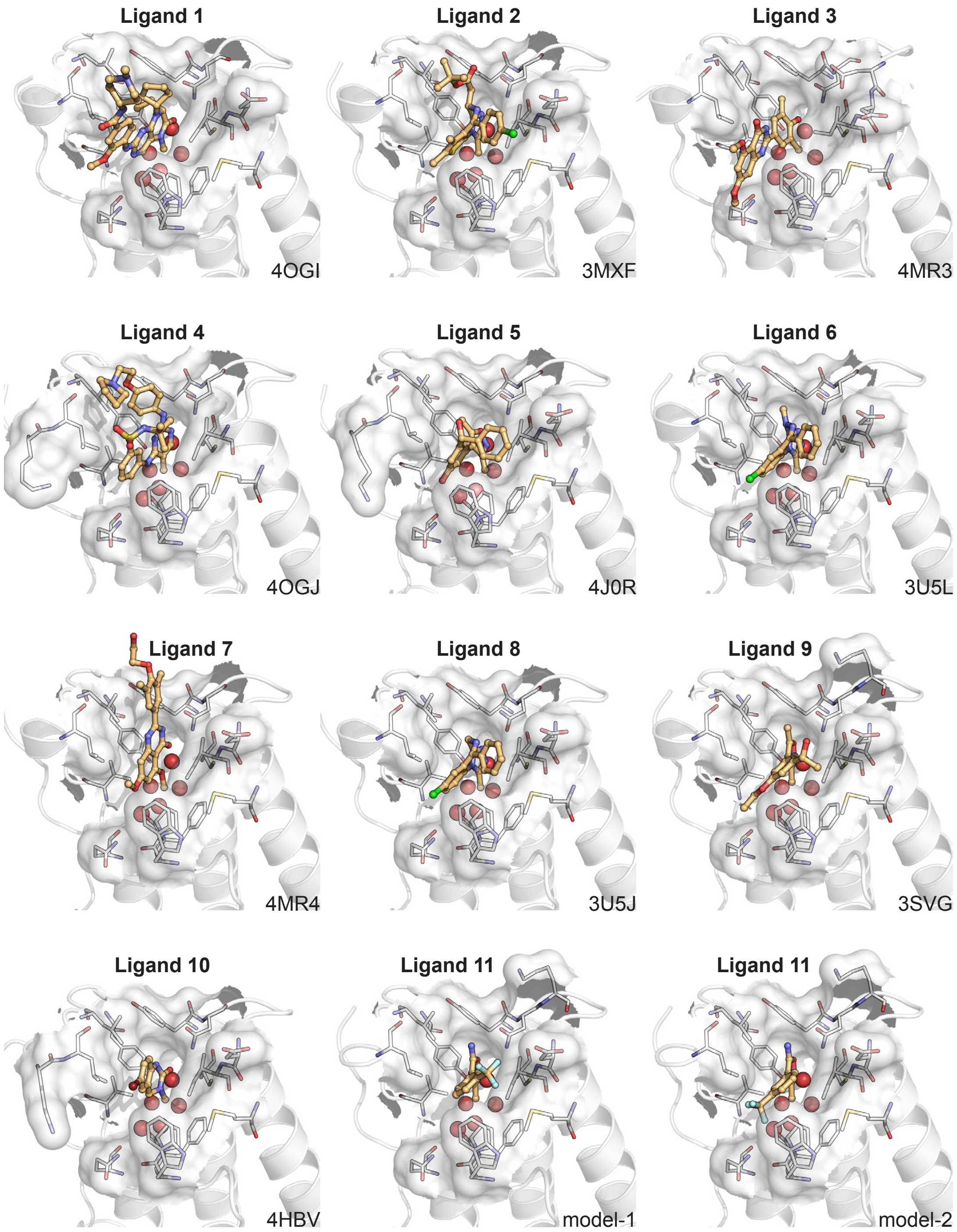

Supplementary Figure 1: X-ray structures of the ligands in complex with BRD4(1). Crystal structures with their reference pdb-ID used as starting conformations for the free energy calculations.

Ligand	$\Delta G^{solv}_{elec+vdw}$	ΔG^{solv}_{restr}	$\Delta G^{prot}_{elec+vdw+restr}$	$\Delta\Delta G_{exp\text{-}lr}$	$\Delta\Delta G_{elec\ corr}$
**1**	189.7 ± 0.2	6.6	-206.6 ± 0.2	-0.7	0.6
	189.7 ± 0.1	6.6	-206.7 ± 0.1	-0.6	0.6
	190.5 ± 0.2	6.6	-207.5 ± 0.3	-0.7	0.6
**2**	59.2 ± 0.2	6.6	-74.8 ± 0.1	-0.7	n/a
	59.4 ± 0.2	6.6	-74.5 ± 0.4	-0.7	n/a
	59.6 ± 0.1	6.6	-74.5 ± 0.1	-0.7	n/a
**3**	219.3 ± 0.1	6.7	-235.1 ± 0.2	-0.6	n/a
	219.1 ± 0.1	6.7	-234.7 ± 0.1	-0.6	n/a
	219.5 ± 0.2	6.7	-233.6 ± 0.1	-0.6	n/a
**4**	444.2 ± 0.3	6.8	-459.8 ± 0.2	-0.8	0.7
	445.1 ± 0.2	6.8	-460.9 ± 0.4	-0.8	0.7
	444.1 ± 0.3	6.8	-460.7 ± 0.2	-0.8	0.7
**5**	57.8 ± 0.1	7.0	-72.7 ± 0.1	-0.5	n/a
	58.0 ± 0.1	7.0	-73.3 ± 0.1	-0.5	n/a
	57.9 ± 0.0	7.0	-73.0 ± 0.1	-0.5	n/a
**6**	24.2 ± 0.1	6.6	-40.6 ± 0.1	-0.6	n/a
	25.4 ± 0.1	6.6	-40.5 ± 0.2	-0.6	n/a
	23.7 ± 0.1	6.6	-40.0 ± 0.1	-0.6	n/a
**7**	201.5 ± 0.1	6.6	-213.0 ± 0.2	-0.5	n/a
	201.4 ± 0.2	6.6	-213.8 ± 0.2	-0.5	n/a
	201.7 ± 0.1	6.6	-213.8 ± 0.2	-0.5	n/a
**8**	4.1 ± 0.1	6.9	-18.5 ± 0.1	-0.5	n/a
	4.4 ± 0.2	6.9	-18.3 ± 0.2	-0.5	n/a
	3.9 ± 0.1	6.9	-18.2 ± 0.2	-0.5	n/a
**9**	57.6 ± 0.1	6.7	-71.8 ± 0.1	-0.4	n/a
	57.3 ± 0.1	6.7	-71.4 ± 0.1	-0.4	n/a
	57.4 ± 0.1	6.7	-71.8 ± 0.2	-0.4	n/a
**10**	89.7 ± 0.1	7.2	-102.6 ± 0.1	-0.4	n/a
	89.6 ± 0.1	7.2	-102.8 ± 0.1	-0.4	n/a
	89.8 ± 0.1	7.2	-102.4 ± 0.1	-0.4	n/a
**11**	57.2 ± 0.1	6.7	-68.3 ± 0.2	-0.4	n/a
	57.2 ± 0.1	6.7	-68.8 ± 0.1	-0.4	n/a
	55.7 ± 0.1	6.7	-66.7 ± 0.1	-0.4	n/a
	55.8 ± 0.1	6.7	-67.1 ± 0.0	-0.4	n/a

Supplementary Table 2: Free energy results starting from the X-ray structures. All values are in kcal/mol. The meaning of the different free energy terms within the thermodynamic cycle is explained in Supplementary Fig. 1. $\Delta\Delta G_{exp\text{-}lr}$ is the contribution to the binding free energy due to the long-range dispersion correction. $\Delta\Delta G_{elec\ corr}$ is the contribution to the binding free energy due to the correction for electrostatic finite size effects and it has been applied to charged ligands only.

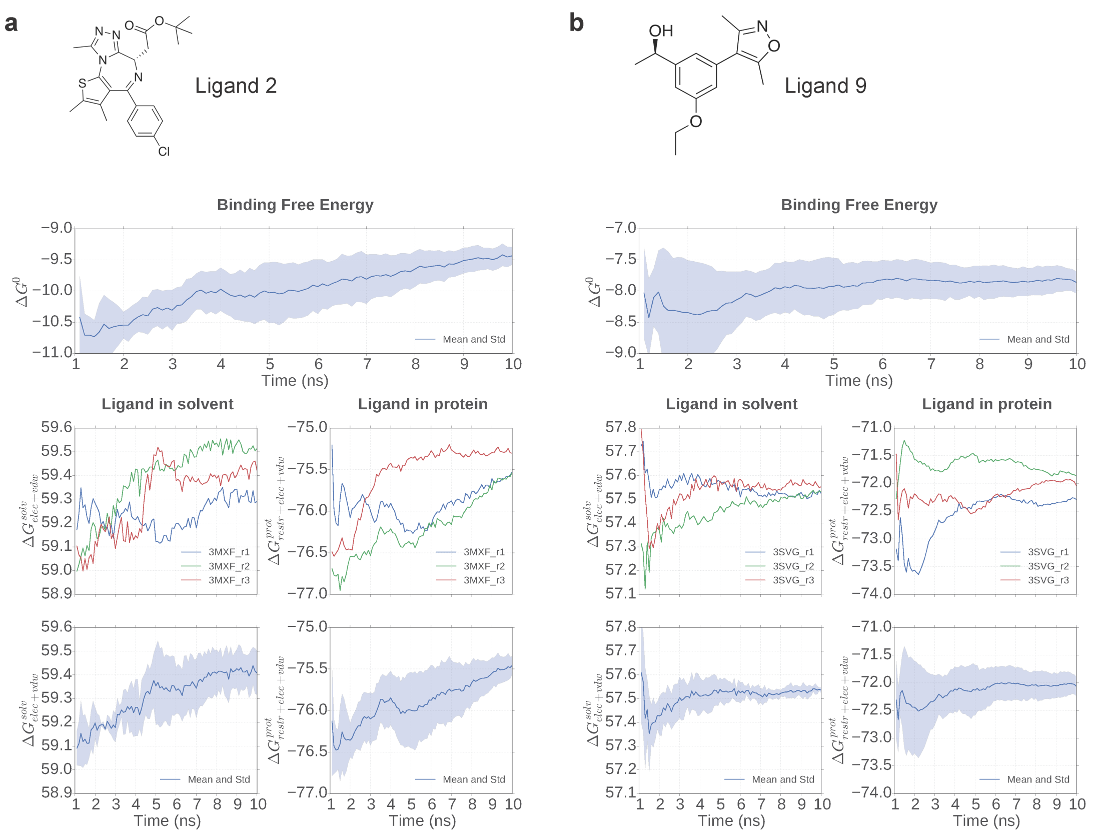

Supplementary Figure 2: Convergence of the calculations. The free energy estimate versus simulated time for the representative examples of (a) ligand 2 and (b) ligand 9 is shown. All the free energies are in kcal/mol. A single MBAR free energy estimate has been carried out every 0.1 ns, starting at 1.1 ns. In the top row is the mean (blue line) and standard deviation (blue shade) of the free energy result ($\Delta G^{0}_{binding}$) for the three replicates. The plots in the middle row show the evolution of $\Delta G^{solv}_{elec+vdw}$ and $\Delta G^{prot}_{restr+elec+vdw}$ for each of the three repeats, while these data are summarized in the bottom row where their mean and standard deviation is shown.

Ligand	Poses	Docking ΔG (kcal/mol)	RMSD to Crystal (Å)
**1**	**a**	-2.69	8.36
	**b**	-2.55	4.14
	**c**	-0.79	3.18
**2**	**a**	-4.67	1.89
	**b**	-1.73	6.80
**3**	**a**	-4.93	6.79
	**b**	-3.52	7.82
	**c**	-2.57	1.99
	**d**	-2.46	3.04
	**e**	-1.57	7.78
**4**	**a**	-3.40	1.80
**5**	**a**	-4.20	5.62
	**b**	-4.18	1.21
**6**	**a**	-4.58	0.96
	**b**	-4.50	4.99
**7**	**a**	-5.18	2.16
	**b**	-3.80	3.88
**8**	**a**	-4.15	0.79
	**b**	-2.36	4.99
**9**	**a**	-4.19	1.75
	**b**	-3.94	4.20
	**c**	-2.11	4.72
**10**	**a**	-3.19	0.69
**11**	**a**	-4.76	n/a
	**b**	-4.44	n/a

Supplementary Table 3: docking results. Shown are the poses retained after the clustering of the results and are ranked by score. Highlighted in orange are the poses that most closely represent the crystallographic binding conformations.

Lig	Ps	$\Delta G^{0}_{binding}$	$\langle \Delta G^{solv}_{elec+vdw} \rangle$	$\Delta G^{solv}_{elec+vdw}$	ΔG^{solv}_{restr}	$\Delta G^{prot}_{elec+vdw+restr}$	$\Delta\Delta G_{exp\text{-}lr}$	$\Delta\Delta G_{elec\ corr}$
**1**	**a**	-10.3 ± 0.8	196.1 ± 0.7	195.6 ± 0.2	6.5	-213.1 ± 0.1	-0.7	0.6
	**b**	-4.6 ± 0.8		197.1 ± 0.3	7.0	-207.8 ± 0.3	-0.6	0.7
	**c**	**-10.9 ± 0.8**		195.7 ± 0.4	6.5	-213.7 ± 0.3	-0.7	0.8
**2**	**a**	**-10.1 ± 0.4**	59.8 ± 0.2	60.0 ± 0.1	6.6	-75.9 ± 0.3	-0.6	n/a
	**b**	-6.7 ± 0.4		59.5 ± 0.0	7.3	-73.2 ± 0.4	-0.5	n/a
**3**	**a**	-6.2 ± 0.2	207.5 ± 0.2	207.5 ± 0.1	6.7	-219.8 ± 0.1	-0.5	n/a
	**b**	-6.5 ± 0.3		207.6 ± 0.1	6.6	-220.1 ± 0.3	-0.4	n/a
	**c**	**-10.8 ± 0.2**		207.6 ± 0.1	6.9	-224.6 ± 0.1	-0.6	n/a
	**d**	-10.5 ± 0.2		207.3 ± 0.1	6.8	-224.3 ± 0.1	-0.5	n/a
	**e**	-7.3 ± 0.2		207.3 ± 0.1	6.8	-221.1 ± 0.1	-0.5	n/a
**4**	**a**	**-9.0 ± 0.8**	443.7 ± 0.3	443.7 ± 0.3	6.7	-461.6 ± 0.3	-0.8	0.6
**5**	**a**	-5.8 ± 0.2	56.5 ± 0.1	56.5 ± 0.1	6.6	-68.5 ± 0.1	-0.4	n/a
	**b**	**-8.3 ± 0.2**		56.6 ± 0.1	6.7	-71.1 ± 0.1	-0.5	n/a
**6**	**a**	-10.3 ± 0.3	24.0 ± 0.1	24.0 ± 0.1	6.6	-40.4 ± 0.3	-0.5	n/a
	**b**	**-10.6 ± 0.3**		24.1 ± 0.1	6.8	-40.8 ± 0.2	-0.6	n/a
**7**	**a**	**-6.6 ± 0.3**	187.1 ± 0.1	187.1 ± 0.1	6.7	-199.9 ± 0.2	-0.5	n/a
	**b**	-5.1 ± 0.1		187.2 ± 0.1	6.7	-198.5 ± 0.1	-0.5	n/a
**8**	**a**	**-10.2 ± 0.2**	4.3 ± 0.1	4.3 ± 0.1	6.6	-20.6 ± 0.1	-0.5	n/a
	**b**	-6.6 ± 0.1		4.3 ± 0.1	6.8	-17.2 ± 0.1	-0.5	n/a
**9**	**a**	**-7.7 ± 0.1**	56.5 ± 0.1	56.4 ± 0.1	6.6	-70.4 ± 0.1	-0.4	n/a
	**b**	-7.3 ± 0.2		56.5 ± 0.1	6.6	-70.0 ± 0.1	-0.4	n/a
	**c**	-6.1 ± 0.1		56.5 ± 0.1	6.8	-69.0 ± 0.1	-0.3	n/a
**10**	**a**	**-6.2 ± 0.1**	89.8 ± 0.0	89.8 ± 0.0	7.1	-102.7 ± 0.1	-0.4	n/a
**11**	**a**	-5.3 ± 0.1	57.0 ± 0.1	57.1 ± 0.0	6.6	-68.6 ± 0.1	-0.4	n/a
	**b**	**-5.4 ± 0.1**		56.9 ± 0.1	6.6	-68.7 ± 0.1	-0.3	n/a

Supplementary Table 4: Free energy results starting from the docking poses. All values are in kcal/mol. Highlighted in orange are the poses that most closely represent the crystallographic binding conformations; in bold are the lowest binding free energies for each ligand. The meaning of the different free energy terms within the thermodynamic cycle is explained in Fig. 1. $\Delta\Delta G_{exp\text{-}lr}$ is the contribution to the binding free energy due to the long-range dispersion correction. $\Delta\Delta G_{elec\ corr}$ is the contribution to the binding free energy due to the correction for electrostatic finite size effects and it has been applied to charged ligands only. All ΔG terms shown here have been rounded to the nearest tenth for ease of reading, despite having been calculated at higher precision.
